# Paravertebral Muscle Mechanical Properties and Spinal Range of Motion in Patients with Acute Neck or Low Back Pain: A Case-Control Study

**DOI:** 10.3390/diagnostics11020352

**Published:** 2021-02-20

**Authors:** Sandra Alcaraz-Clariana, Lourdes García-Luque, Juan Luis Garrido-Castro, César Fernández-de-las-Peñas, Cristina Carmona-Pérez, Daiana Priscila Rodrigues-de-Souza, Francisco Alburquerque-Sendín

**Affiliations:** 1 Department of Nursing, Pharmacology and Physical Therapy, Faculty of Medicine and Nursing, University of Córdoba, 14004 Córdoba, Spain; m72alcls@uco.es (S.A.-C.); z12galul@uco.es (L.G.-L.); falburquerque@uco.es (F.A.-S.); 2 Department of Computer Science and Numerical Analysis, Rabanales Campus, University of Córdoba, 14071 Córdoba, Spain; cc0juanl@uco.es; 3 Maimonides Biomedical Research Institute of Cordoba (IMIBIC), 14004 Córdoba, Spain; 4 Department of Physical Therapy, Occupational Therapy, Rehabilitation and Physical Medicine, Universidad Rey Juan Carlos, 28922 Alcorcón, Spain; cesar.fernandez@urjc.es; 5 Cátedra Institucional en Docencia, Clínica e Investigación en Fisioterapia: Terapia Manual, Punción Seca y Ejercicio Terapéutico, Universidad Rey Juan Carlos, 28922 Madrid, Spain; 6 Centro de Recuperación Neurológica de Córdoba (CEDANE), 14005 Córdoba, Spain; mcarperes@yahoo.es

**Keywords:** myotonometry, kinematics, cervical spine, low back pain, neck pain

## Abstract

Our aims were to identify potential differences in muscle mechanical properties (MMPs) of cervical and lumbar tissues and in spinal range of motion (ROM) between patients with acute low back pain (LBP) or acute neck pain (NP) and healthy controls, and to identify if ROMs and MMPs are able to identify subjects among the three groups. Clinical variables (pain, disability, fear of movement, kinesiophobia, quality of life), MMPs and ROMs were obtained in 33 subjects with acute LBP, 33 with acute NP, and 33 healthy control subjects. Between-groups differences and explanatory models to discriminate groups depending on MMPs and ROMs were calculated. The results showed that cervical tone was higher in patients with acute NP than in controls, while cervical decrement was higher in both spinal pain groups. Patients with acute NP showed reduced cervical flexion when compared to acute LBP and control groups, and also cervical rotation, but just against controls. Furthermore, lumbar flexion was reduced in patients with acute LBP when compared to those with acute NP. Cervical decrement was able to discriminate spinal pain individuals from controls in a multinominal regression (R^2^: Cox–Snell estimation = 0.533; Nagelkerke estimation = 0.600). Lumbar flexion differentiated patients with acute LBP and controls, whereas cervical flexion differentiated patients with acute NP and controls. This study supports a tendency of the affectation of other spinal regions when only one is affected.

## 1. Introduction

Low back pain (LBP) is associated with substantial burden at individual level and health systems [1]. In fact, it is the main cause of years lived with disability [2]. Subjects suffering LBP show spinal reduced movement and smaller amplitude [3,4], differences in muscle size, muscle tone, stiffness, or fat infiltration [5,6,7], muscle weakness [8,9] or disturbed recruitment patterns [3,10]. All these features and their relations with psychosocial domains have been well studied in chronic LBP [11,12,13], but not enough information is available in acute LBP.

Neck pain (NP) is the fourth largest contributor to global disability worldwide [14]. Individuals with NP show limited range of motion (ROM) [15], pain adaptive motor control disturbances, such as increased activation of surface musculature [15,16], and modifications in muscle mechanical properties (MMPs) [16,17]. However, these disturbances are not completely understood [18] and most of these conditions are diagnosed and classified as unspecific [19].

According to the biopsychosocial model, both LBP and NP are influenced by multiple factors, e.g., psychological aspects like catastrophism, patient’s beliefs and expectations, physical activity, environmental, genetics, morphological or mechanical [13,20], being some of them predictors of chronicity [19,21,22,23]. Better understanding of this predictors and their clinical course [24,25], in combination with other measures, such as MMPs and ROMs, are necessary for establishing treatment strategies in clinical practice.

Some technologies have improved the characterization of spinal disorders. With respect to muscle tissue state, few studies have focused on the MMPs of the paravertebral muscles [26]. These features have usually been assessed by using subjective methods [27], such as palpation. Myotonometry is a recent innovative technology, which provides reliable data on MMPs in clinical environments [28]. Furthermore, preliminary studies suggest that muscular injuries have their own distinctive MMPs, assessed with myotonometry, which can help to understand muscle deficits related to injury [28]. These changes in lumbar MMPs have been observed in adults with ankylosing spondylitis [29], but current evidence analyzing lumbar MMPs in individuals with LBP is restricted to chronic state [30]. In the cervical region, it has been suggested that factors such as pain and disability could be responsible for an increase in tone and stiffness in spinal pain populations [31].

The use of new technologies could be also applied for an objectification of changes in ROM, both in the lumbar [32,33] and cervical [34] regions. For instance, inertial motion units (IMUs) are small, cheap, accurate, reliable, and easy to apply in clinical practice [35]. These sensors have been validated for cervical, lumbar and hip analyses [36,37] and have been able to differentiate the lumbo-pelvic kinematics between healthy and back pain populations [3], and to classify subtypes of traumatic injuries in the cervical spine [38]. However, few studies have focused on the relationship between the kinematics of the neck or lower back with clinical scales [39], and most of them have focused only on chronic spinal pain populations [33,38,39].

Thus, the primary objective of this study was to identify differences in MMPs of cervical and lumbar tissues, as assessed with myotonometry, and in cervical and lumbar ROMs, as assessed with IMUs, between patients with acute LBP or acute NP and healthy subjects. Second, this study tried to identify if ROMs and MMPs are able to correctly identify subjects among the three groups, and to assess the relations between MMPs, ROMs, sociodemographic and clinical data.

## 2. Methods

### 2.1. Study Design

An observational, cross-sectional case-control study with consecutive sampling was conducted. Participants with acute LBP or acute NP at the moment of the evaluation were recruited through a non-probabilistic sampling from three centers, Physiobalance (a private physiotherapy center), Reina Sofía University Hospital of Córdoba (Andalusian Health Service), and the Biosanitary campus of the University of Córdoba, in Spain, from November 2018 to January 2020. For improving the comparability among groups, by each individual with acute NP included in the study, an individual with acute LBP, and a healthy subject, matched by age (±3 years), body mass index (BMI) (±2 Kg/m^2^) and sex (maximum difference in sex distribution among groups: 4 individuals) was also recruited. This project was approved by the Cordoba Research Ethics Committee (registration number 4017/2018). All participants signed a written informed consent.

### 2.2. Participants

We included two different case groups. One group formed by subjects of both sexes aged from 18 to 65 years old, who presented acute NP (<4 weeks evolution [40]), and pain ≥3 score assessed with a numerical pain rate scale (NPRS) [41]. A second group was composed by individuals with acute LBP who met the inclusion criteria described above. Participation in the study was proposed to those subjects who requested assistance at the study centers and met the selection criteria. The third group was the control group and included subjects of both sexes comparable in age, recruited by local advertising at the study centers, with no spinal pain symptoms in the previous 6 months. The exclusion criteria were common for all groups and included: traumatic history, spine surgery, congenital deformity, inflammatory disease, pregnancy, received physiotherapy treatment for the spine in the last 6 months.

### 2.3. Sample Size

Sample calculation was performed by using the G*Power 3.1 software with the analysis of variance (ANOVA) one way (F-test) as a statistical test. To achieve a moderate *f* effect size of 0.33, which is common in clinical practice for musculoskeletal outcomes [42], for MMPs or ROM outcomes, with an α coefficient of 0.05 and a power of 0.80, 30 individuals per group are necessary. Finally, 33 individuals per group were included, due to possible missing data.

### 2.4. Assessments and Procedures

Several questionnaires commonly applied in clinical settings were used to identify pain behaviors and beliefs about pain, disability and general health (see next section). In addition, sociodemographic aspects, e.g., age, sex, weight, height, BMI were also collected. Cervical and lumbar spine ROMs and MMPs were finally assessed. The entire evaluation lasted approximately 45 min.

#### 2.4.1. Assessment of Muscle Mechanical Properties (MMPs)

A record of the MMPs using a hand myotonometry (MyotonPro^®^, Estonia) was made in both lumbar and cervical spines. The MyotonPro^®^ provides a controlled preload of 0.18 N for an initial compression of the subcutaneous tissue, imposing an additional 15 ms pulse and 0.40 N of mechanical force, which induces a natural damped oscillation in the targeted tissue. This response is measured by an accelerometer [43]. The MMPs recorded in this study included: *frequency*, measured in Hz, representing the muscle tone at rest (the higher frequency, the higher muscle tone); *stiffness*, measured in N/m, reflecting the capacity of the muscle to resist contraction or external pressure to deform (the greater stiffness, the greater muscle toughness); logarithmic *decrement* of oscillation amplitude, that has no unit, and is a measure of muscle elasticity (the higher decrement, the lower elasticity [44]); *creep*, that has also no unit, the material property in which progressive deformation occurs with time while a constant stress is applied; and, *relaxation*, measured in ms., describing the phenomenon of stress decrease with time, while the applied strain is constant, being the stress relaxation time the recovery time for the material to return to its normal state after deformation [45].

**Figure 1:**
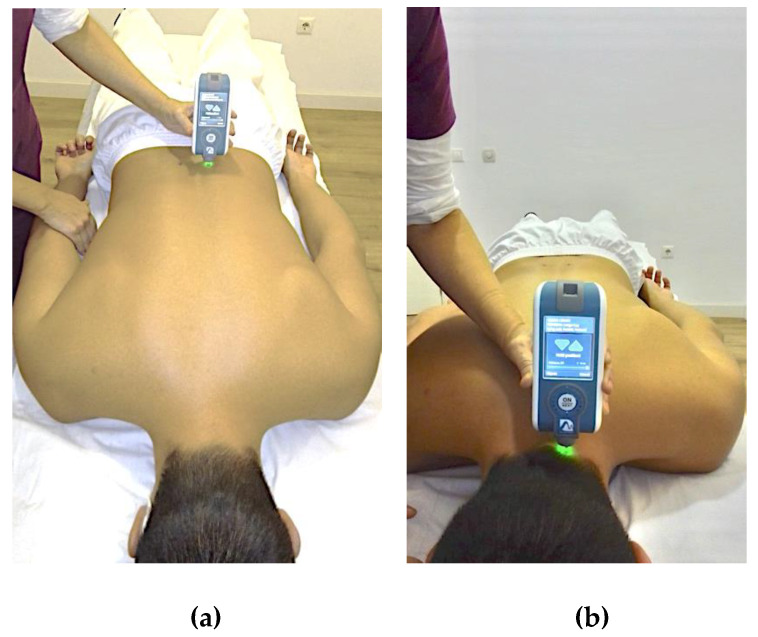
Assessment of muscle mechanical properties (MMPs). (**a**) Lumbar myotonometry. Subject position and device location. (**b**) Cervical myotonometry. Subject position and device location.

For data collection, subjects were placed in a prone position with both arms along the body. They were asked to hold apnea for 5 s after exhalation to reduce abdominal influence on the test. The cervical and low back regions were exposed throughout the procedure. The probe was first loaded by pushing against the skin surface to the required depth (indicated by a change in light from red to green), and the device applied impulses to induce damped oscillations within the muscle belly. During the test, the coefficient of variation (CV) of each test result was observed, and if the CV was more than 3%, the test was repeated again [44]. Lumbar measurements were carried out by placing the probe of the device perpendicular to the muscular belly of the erector spinal column, 2.5 cm from the midline of the spinous process of L5 [46] (Figure 1a). For the cervical measurements, the semispinalis capitis muscles at C4 level were assessed [31,47] (Figure 1b). The order of assessments (right/left) was randomized by a randomization plan generator (www.randomization.com, accessed on 10 October 2018). The evaluations of the first 10 subjects of each group was repeated after one week to assess between days, intra-rater reliability. The intraclass correlation coefficient (ICC) was >0.8 for all assessments and variables. Since no side-to-side differences in acute LBP, acute NP or control groups were observed, the mean of both sides was considered in the main analysis.

#### 2.4.2. Range of Motion (ROM) Assessment

**Figure 2:**
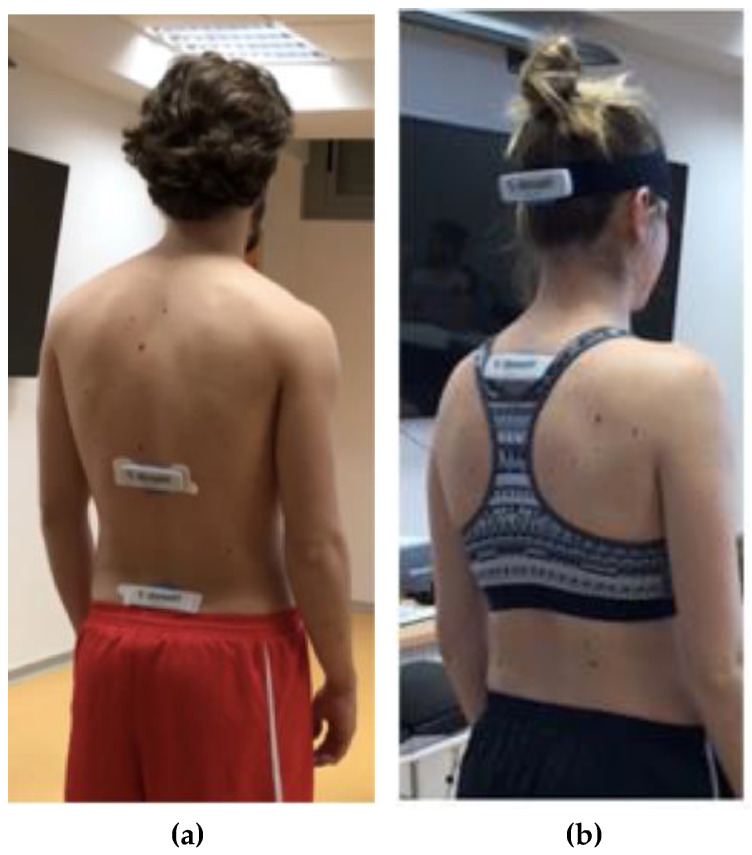
Range of motion (ROM) assessment with inertial motion units (IMUs). (**a**) Lumbar assessment. Subject position and device location. (**b**) Cervical assessment. Subject position and device location.

For the analysis of the spinal mobility, IMUs (Dosarvi ViMoveTM^®^) were used. The evaluation was performed always after myotonometry. Using two IMUs, the range of the different movements of the lumbar and cervical spine was recorded [48]. In the lumbar spine evaluation, one of the IMUs was placed on the line that joins the posterior-superior iliac spines and, depending on the height of the subject, a template provided by the ViMove system was used to identify the location of the second sensor (Figure 2a).

The movements were first explained and demonstrated by the assessor prior to the evaluation. After asking the subject to remain in a neutral position for 5 s, the following sequence for the ROM assessment was conducted: lumbar flexion, lumbar extension, lumbar lateral-flexion (full range adding left and right lateral-flexions), and lumbar rotation (full range adding left and right rotations). For the cervical evaluation, the sensors were placed on the occiput of the patient using a strap, and 10 cm below C7 vertebra (Figure 2b). With the same methodology as in the lumbar region, the sequence was as follows: cervical flexion, cervical extension, cervical lateral-flexion (full range), and cervical rotation (full range).

A total of 3 repetitions of each movement were executed, up to the maximum possible range without rebounding and without pain [48]. The validity and reliability of these procedures in clinical setting has been confirmed in previous studies [3].

#### 2.4.3. Self-Reported Questionnaires

The Spanish Version of the McGill Pain Questionnaire was used to determine the intensity and dimensions of pain. This questionnaire consists of 66 descriptors distributed in 19 subclasses each to describe pain to address a total of three dimensions (sensory, affective and evaluative) and a visual analogue scale [49,50]. The Pain Rating Index (PRI) total and for each of the dimensions (calculated by adding the score for each group of words that make up each category) and the number of words chosen (NWC) were considered in the current study. The validity and reliability of the Spanish version of the McGill Pain Questionnaire respects to the original version have been found to be high showing correlations with the original scale ranging from 0.89 to 0.98 [51].

The Oswestry Disability Index (ODI) evaluates disability due to LBP [52]. It consists of 10 questions with 6 answer possibilities, each about basic activities of daily life that can be affected by pain, scoring from 0 (no disability) to 5 (highest disability). The total score is calculated in percentage and interpreted as follows: 0–20%: minimal disability; 21–40%: moderate disability; 41–60%: severe disability; 61–80%: crippling back pain; 81–100% (these patients are either bed-bound or have an exaggeration of the symptoms) [53]. The Spanish version of ODI has demonstrated good internal consistency (Cronbach’s α = 0.92) and good construct validity in patients with acute LBP [54].

The Neck Disability Index (NDI) was applied to assess neck pain related-disability [55]. It is composed by 10 sections (intensity of pain, personal care, weight lifting, reading, headache, ability to concentrate, ability to work, driving of vehicles, sleep and leisure activities) with 6 possible answers scored from (no disability) to 5 (highest disability). The total score is expressed in percentage as follows: 0–8%: no disability; 10–28%: mild disability: 30–48%: moderate disability; 50–64%: severe disability; 70–100%: complete disability [56]. The Spanish version used in our study has demonstrated reasonable validity, consistency, reliability and sensitivity to change, with a Cronbach’s alpha of 0.89 and an ICC of 0.88 [57].

The Fear-Avoidance Beliefs Questionnaire (FABQ) and the Short Version of Tampa Scale for Kinesiophobia (TSK-11SV) were used in both groups to assess pain behavior, because fear-avoidance or kinesiophobia cover different conceptual definitions. Kinesiophobia refers to fear of movement that results from a pain vulnerability, while fear-avoidance refers to the avoidance of a potential threat with physiological, cognitive, and behavioral responses, which leads to potential fear [58]. The FABQ [59,60] consists of 16 phrases related to physical activity (first 5 items), which composes the physical activity subscale (FABQ-PA), and work (last 11 items), which composes the work subscale (FABQ-W). Each question range is from 0 (totally disagree) to 6 (totally agree) [61]. The total score range is from 0 to 96, with a higher value reflecting a higher degree of fear-avoidance beliefs of pain. The Spanish version has shown high internal consistency (Cronbach’s α = 0.93) [61] and test-retest reliability (FABQ-W r = 0.91; FABQ-PA r = 0.84) [62]. The TSK-11SV [60] has shown satisfactory psychometric properties for musculoskeletal pain conditions including LBP and NP [63,64]. In this study, the Spanish version of the TSK-11SV was used consisting of 11 items related to the somatic approach and avoidance of activity. Each item is rated on a four-point scale ranging from “strongly agree” to “strongly disagree”. Total score ranges from 11 to 44 with higher scores indicating more fear of movement and/or injury recurrence. The internal consistency is satisfactory for patients with acute pain (Cronbach’s α = 0.81) and the stability between measurements is moderate (Pearson’s r 0.55) [65].

The 12-Item Short-Form Health Survey (SF-12), divided by the physical component (PCS-12) and the mental component (MCS-12), was used to assess the health-related quality of life [66,67]. Each of the 12 items has the possibility of 3 to 5 answers, with lower values indicating poorer health-related quality of life on a scale of 0 to 100. The SF-12 has shown good internal consistency (Cronbach’s α from 0.72 to 0.89) and test-retest reliability (ICC from 0.73 to 0.86) [68]. Regarding country-specific validity, high correlations (ICC = 0.94) between Spain SF-12 and SF-36 were found [66].

### 2.5. Statistical Analysis

Categorical variables are presented as counts and percentages. Continuous data were described by mean and standard deviation with a 95% confidence interval (95% CI). The Kolmogorov–Smirnov test showed their normal distribution (all variables: *p* > 0.05).

For the primary aim of the study, when the three groups were compared, one-way ANOVAs, with Tukey test for post hoc analyses, were conducted. When only spinal pain groups were included in the analysis, as occurred with fear of pain and kinesiophobia questionnaires, the unpaired Student *t*-test were applied.

To determine if each MMP of spinal musculature and each spinal ROM can classify individuals between the three groups, receiver operating characteristics (ROC) curves were developed. To interpret them, statistical significance and the area under the curve (AUC) were calculated. For the AUC, a value of 0.5 was considered fail to discriminate, 0.6–0.7 was considered poor, 0.7–0.8 was considered acceptable, 0.8–0.9 was considered excellent, and outstanding when more than 0.9 [69].

Furthermore, to determine the influence of the MMPs and the ROMs on suffering NP or LBP, with control group as the state pattern, a multi-nominal regression analysis was applied, with MMPs and ROMs as potential predictor variables, and the clinical state (LBP, NP, or control) as the dependent variable. Age, sex and BMI were also tested as predictors. The R^2^ estimation was calculated with Cox–Snell and Nagelkerke tests, and odds ratios (OR) for each factor were also reported. Finally, the associations of each factor were considered as meaningful when statistically significant at *p* < 0.05 was observed. Percentages of correctly classified individuals according to the model were presented.

For the secondary objectives, to identify intra-group associations among the outcomes and other sociodemographic and clinical features, Pearson *r* coefficients were calculated. Correlations were considered as negligible (0.0 to 0.19), fair (0.20 to 0.39), moderate (0.40 to 0.69), strong (0.70 to 0.89) or almost perfect (0.0 to 1.00) [70].

For all tests, the level of significance was set at 0.05, and the IBM-SPSS^®^, version 25 (Armonk, NY, USA) was used for the statistical analysis.

## 3. Results

### 3.1. Differences in MMPs and ROMs among Groups

Table 1 shows the scores in all outcomes of the three groups. Age, sex, BMI and MCS-12 were not different among the three groups. The PCS-12 was different between both spinal pain groups and the control group with more than 10 points higher for healthy controls. Additionally, pain intensity, assessed with NPRS, NWC, PRI-total, FABQ scores, and TSK-11SV did not show statistical differences between the subjects with spinal pain (acute LBP or acute NP).

The MMPs showed significant differences among groups for cervical tone and cervical decrement: cervical tone was significantly higher (*p* = 0.048) in the acute NP group (1.1 Hz, 95% CI 2.3–0.1) than in the control group, but not significantly different than the LBP group (0.6 Hz, 95% CI −0.6–1.9). Cervical decrement was significantly higher (*p* = 0.001) in both acute NP and acute LBP groups than in healthy controls (mean differences 0.19, 95% CI 0.06–0.31 and 0.17, 95% CI 0.29–0.04, respectively). No other significant differences among groups in the remaining MMPs outcomes were observed (Table 1).

Within cervical ROMs, cervical flexion was significantly reduced (*p* = 0.010) in the acute NP group compared to both acute LBP (5.8°, 95% CI 11.0–0.7) and control (5.7°, 95% CI 10.9–0.6) groups; whereas cervical rotation was significantly (*p* = 0.047) reduced in the acute NP group as compared to controls (12.1°, 95% CI 25.0–0.3). No differences in cervical lateral-flexion ROM was found (Table 1). Furthermore, lumbar ROM only showed significant differences for lumbar flexion (*p* = 0.024), showing significant reduced mobility in the acute LBP group as compared to the acute NP group (8.1°, 95% CI 15.3–0.9). There were no significant differences among the groups for lumbar lateral-flexion and rotation (Table 1).

### 3.2. Receiver Operating Characteristics (ROC) Curves Based on MMPs and ROMs and Multinomial Regression

**Figure 3:**
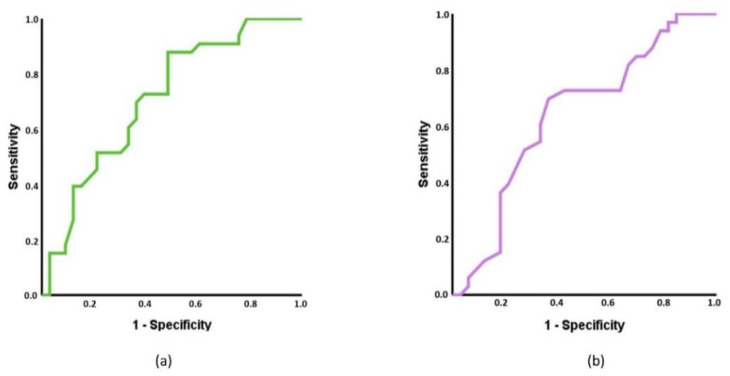
Receiver operating characteristic (ROC) curve of the cervical decrement (**a**) and lumbar flexion (**b**), to discriminate between individuals with acute low back pain and controls. (**a**) Area under the curve (AUC): for cervical decrement = 0.709 (95% confidence interval (CI) = 0.584–0.835). (**b**) AUC: lumbar flexion = 0.660 (95% CI = 0.524–0.791).

**Figure 4:**
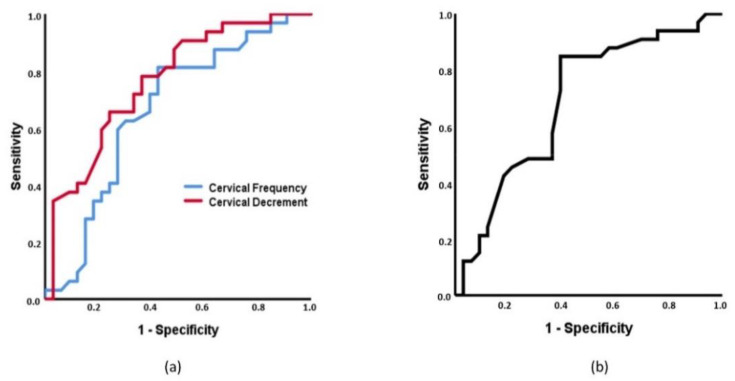
Receiver operating characteristic (ROC) curve of the cervical tone and decrement (**a**) and cervical flexion (**b**), to discriminate between individuals with acute neck pain and controls. (**a**) Area under the curve (AUC): for cervical tone = 0.663 (95% CI = 0.527–0.798); for cervical decrement = 0.764 (95% CI = 0.648–0.879). (**b**) AUC: cervical flexion = 0.691 (95% CI = 0.560–0.821).

To subclassify individuals with acute LBP and healthy controls, cervical decrement and lumbar flexion achieved statistically significance with an AUC of 0.709 (95% CI 0.584–0.835, *p* = 0.003), Figure 3a) and 0.660 (95% CI 0.524–0.791, *p* = 0.047, Figure 3b), respectively. To classify individuals with acute NP vs. controls, cervical tone, cervical decrement and cervical flexion showed statistical significance with an AUC of 0.663 (95% CI 0.527–0.798, *p* = 0.024), 0.764 (95% CI 0.648–0.879, *p* < 0.001, Figure 4a), and 0.691 (95% CI 0.560–0.821, *p* = 0.008, Figure 4b), respectively.

When sociodemographic, MMPs, and ROM were included in a multinomial regression for predict patients with NP or acute LBP with respect to healthy controls as reference state, a statistically significant model with moderate to high R^2^ (Cox–Snell estimation: 0.533; Nagelkerke estimation: 0.600) was identified (*p* = 0.001). The variables involved in the model for LBP patients were cervical lateral-flexion (OR 1.087, 95% CI 1.001–1.177, *p* = 0.049), cervical decrement (OR 7.153, 95% CI 2.872–11.028, *p* = 0.02), and lumbar tone (OR 0.509, 95% CI 0.253–0.992, *p* = 0.048). Thus, individuals with acute LBP were more likely to exhibit higher cervical lateral-flexion and cervical decrement and lower lumbar tone. The variables involved in the model for acute NP individuals were lumbar (OR 1.087, 95% CI 1.003–1.175, *p* = 0.048) and cervical (OR 0.897, 95% CI 0.811–0.992, *p* = 0.033) flexion and cervical decrement (OR 8.002, 95% CI 3.330–14.476, *p* = 0.01), that is, patients with acute NP were more likely to exhibit reduced lumbar and cervical flexion ROM and higher cervical decrement. No other factor was associated to the individual pain state. The model was able to correctly identify 67.3% of the patients with better results for acute NP and healthy controls (71.9%) than for identifying acute LBP (57.6%) (Table 2).

### 3.3. Intra-Group Associations among Outcomes, Sociodemographic, and Clinical Variables

In the acute LBP group, age showed a defined pattern, being negatively correlated with lumbar flexion (r = −0.547, *p* = 0.001) and lateral-flexion (r = −0.397, *p* = 0.022) ROM and with all cervical spine ROMs (flexion: r = −0.545, *p* = 0.001; extension: r = −0.460, *p* = 0.007; rotation: r = −0.352, *p* = 0.045; lateral-flexion: r = −0.533, *p* = 0.001) and positively correlated with muscle tone (lumbar: r = 0.554, *p* = 0.001; cervical: r = 0.342, *p* = 0.048) and decrement (r = 0.565, *p* = 0.001; cervical: r = 0.687, *p* < 0.001), and stiffness of lumbar muscles (r = 0.555, *p* = 0.001). In addition, age was negatively correlated with relaxation of lumbar muscles (r = −0.455, *p* = 0.008). The BMI was negative and moderately correlated with cervical extension (r = −0.421, *p* = 0.015) and lateral-flexion (r = −0.400, *p* = 0.025) ROM and with several cervical MMPs such as tone (r = 0.416, *p* = 0.016), stiffness (r = 0.436, *p* = 0.011), relaxation (r = 0.468, *p* = 0.006) or creep (r = 0.455, *p* = 0.008). Pain intensity (NPRS) showed positive associations with lumbar tone (r = 0.471, *p* = 0.006) and stiffness (r = 0.365, *p* = 0.037) and also with cervical decrement (r = 0.423, *p* = 0.014). The SF-12 domains, ODI or kinesiophobia questionnaires were not significantly correlated with MMPs or ROMs. Finally, lumbar flexion was negative and moderately associated with the FABQ total score (r = −0.401, *p* = 0.020).

For the relationships between ROM and MMPs, only lumbar flexion and cervical rotation showed correlations with MMPs. Thus, lumbar flexion was negatively correlated with lumbar tone (r = −0.372, *p* = 0.033) and stiffness (r = −0.359, *p* = 0.040) and cervical decrement (r = −0.399, *p* = 0.024). Finally, cervical rotation was correlated with lumbar MMPs, being negatively correlated with tone (r = −0.529, *p* = 0.002), stiffness (r = −0.534, *p* = 0.001) and decrement (r = −0.386, *p* = 0.026), and positively with relaxation (r = 0.405, *p* = 0.019) and creep (r = 0.352, *p* = 0.045, Table 3).

In the acute NP group, age also showed negative and moderate correlations with lumbar flexion (r = −0.459, *p* = 0.007) and lateral-flexion (r = −0.401, *p* = 0.020) and cervical extension (r = −0.512, *p* = 0.002), lateral-flexion (r = −0.674, *p* < 0.001) and rotation (r = −0.483, *p* = 0.004). Furthermore, age was also positively correlated with lumbar (r = 0.458, *p* = 0.008) and cervical (r = 0.541, *p* = 0.001) decrement. In addition, BMI was negative and moderately correlated with lumbar flexion (r = −0.388, *p* = 0.026) and lateral-flexion (r = −0.423, *p* = 0.014), and with cervical flexion (r = −0.403, *p* = 0.020) and extension (r = −0.513, *p* = 0.002). The BMI showed fair correlations with lumbar relaxation (r = 0.351, *p* = 0.047) and lumbar (r = 0.379, *p* = 0.033) and cervical (r = 0.349, *p* = 0.049) creep. Pain intensity (NPRS) was negatively correlated with cervical rotation (r = −0.433, *p* = 0.012) and NWC and sensory PRI were negatively correlated with cervical decrement (r = −0.375, *p* = 0.034) and cervical flexion (r = −0.402, *p* = 0.021), respectively. The SF-12 did not show any correlation with any MMP or ROM. The NDI was negatively correlated with cervical lateral-flexion (r = −0.413, *p* = 0.017) and rotation (r = −0.504, *p* = 0.003) and with lumbar extension (r = −0.376, *p* = 0.031). No significant correlation with MMPs was observed. Fear to movement and kinesiophobia did not exhibit correlations with MMPs or ROM, with the exceptions of FABQ-PA, that showed a positive and moderate correlation with cervical decrement (r = 0.401, *p* = 0.026) and with the FABQ-W, that was associated with lumbar stiffness (r = −0.367, *p* = 0.042) and relaxation (r = 0.416, *p* = 0.020).

For the relationships between ROMs and MMPs, cervical flexion was negatively correlated with lumbar creep (r = −0.409, *p* = 0.020), while cervical lateral-flexion was negatively associated with lumbar (r = −0.522, *p* = 0.002) and cervical (r = −0.364, *p* = 0.041) decrement. For lumbar movements, only lumbar lateral-flexion showed significant correlation with lumbar decrement (r = −0.545, *p* = 0.001) and creep (r = −0.348, *p* = 0.049, Table 4).

Finally, in the control group, age was highly correlated with all lumbar MMPs (tone: r = 0.644; *p* < 0.001; stiffness: r = 0.598, *p* < 0.001; decrement: r = 0.653, *p* < 0.001; relaxation: r = −0.629, *p* < 0.001; creep: r = −0.410, *p* = 0.021) and with cervical decrement (r = 0.529; *p* = 0.002). Furthermore, age was also negatively associated with lumbar flexion (r = −0.376, *p* = 0.031) and extension (r = −0.650, *p* < 0.001) and with cervical extension (r = −0.354, *p* = 0.043). The BMI was negatively correlated with lumbar flexion (r = −0.367, *p* = 0.035) and lateral-flexion (r = −0.459, *p* = 0.007), and cervical extension (r = −0.502, *p* = 0.003). Furthermore, the BMI showed positive and moderate correlations with cervical relaxation (r = 0.494, *p* = 0.003) and creep (r = 0.510; *p* = 0.002). As occurred with the spinal pain groups, the SF-12 showed no relation with any MMPs or ROM outcome.

With respect to MMPs and ROM, the relationships showed a consistent pattern, where lumbar flexion (tone: r = −0.411; *p* = 0.017; stiffness: r = −0.341, *p* = 0.048; decrement: r = −0.384, *p* = 0.027; relaxation: r = 0.413, *p* = 0.017; creep: r = 0.341, *p* = 0.048) and extension (tone: r = −0.354; *p* = 0.040; stiffness: r = −0.381, *p* = 0.029; decrement: r = −0.490, *p* = 0.004; relaxation: r = 0.629, *p* < 0.001; creep: r = 0.570, *p* = 0.001) were associated to all lumbar MMPs and also to cervical decrement (r = −0.401; *p* = 0.021). Furthermore, cervical extension was fairly associated with cervical creep (r = −0.352, *p* = 0.045), and cervical lateral-flexion with lumbar decrement (r = −0.464, *p* = 0.007, Table 5).

## 4. Discussion

The current study showed that specific MMPs, such as cervical tone and elasticity, and cervico-lumbar flexion and cervical rotation ROMs are different among patients with acute LBP, acute NP and healthy controls. Furthermore, no lumbar MMP was able to differentiate groups, but the reduction of flexion on each affected region and cervical elasticity allowed to discriminate individuals between spinal pain and healthy controls. In general, the MMPs and ROMs were not associated with disability or behavior and quality of pain within the spinal pain groups, while age was related to a reduction in ROMs and an increase of tone, stiffness and decrement, and a reduction of relaxation and creep, in both lumbar and cervical regions in all groups. Lumbar ROM in the sagittal plane was inversely related to tone, stiffness, and decrement in healthy controls, being absent this pattern in the cervical region, and in patients with acute NP. Unexpectedly, cervical rotation was inversely related to lumbar tone, stiffness, and decrement, and directly to relaxation and creep in people with acute LBP. This pattern, where cervical values are associated in patients with acute LBP, again appeared when the capacity of discrimination of MMPs and ROMs among groups was tested, supporting the influence of a specifically located spinal mechanical pain along other regions of the spine.

The whole protocol was applied without unexpected interruptions or the appearance of pain during the examination, which reinforce the clinical applicability of the collection of ROMs assessed by IMUs, and of MMPs assessed with MyotonPro^®^.

### 4.1. Differences in MMPs and ROMs between Spinal Pain and Controls

Some cervical MMPs and lumbar and cervical ROMs were different among the study groups. These differences distinguished not only healthy controls from individuals on each spinal involved region, as occurred with high cervical tone and low cervical rotation ROM within the acute NP group, but also for the non-affected regions, such as the high cervical decrement in patients with acute LBP.

The mean values of lumbar musculature stiffness of young control subjects [28] and elder control subjects [44] were similar to those values obtained in the current study for all groups. Furthermore, current values of tone and stiffness were lower than those reported for other pathological populations, such as young [28,71] and elderly [44] individuals with chronic spinal pain, and even inflammatory pain [29]. In fact, the differences among the three groups, which did not show statistical significance, were lower than the minimum detectable change established for these variables in the lumbar muscles [29], and may be explained by the acute state of patients in our study, and the rest assessment position. By contrast, the altered pattern of greater stiffness and lower elasticity appeared for the cervical region. For this region, it has been described that the stiffness of the splenius capitis decreased and the elasticity increased after the administration of botulinum neurotoxin injections in patients with cervical dystonia [27]. In the study, the baseline values were higher than those obtained in our study, but the final ones can be considered similar, mainly for the control subjects, being tone and decrement higher in spinal pain individuals, which confirms a moderate alteration of the MMPs in the cervical region for acute LBP and NP groups.

Some authors have identified decreased lumbar ROMs in LBP patients [3], and specifically in the lumbar flexion in chronic patients [3], this movement being the most studied one at lumbar level. This was the case of the current data, with absolute values of lumbar flexion ROM being similar to those previously identified for LBP (≈53°) and controls (≈47°) [3,72,73], which exceeds the minimum detectable change for chronic LBP [36]. Other ROMs, such as lumbar extension or lateral-flexion did not show between-groups differences in our study, and the absolute values were similar in means, than other previously reported, independent of the use of ViMove system [72] or not [36]. Scarce studies have measured lumbar rotation due to technical limitations [36,72], which limits the possibility of comparisons.

For the cervical measurements, the ROM found in patients with acute NP was similar to previously reported data in healthy controls [74] and lower than the values of patients with acute LBP or the control individuals in our study, which could be interpreted as a compensatory mechanism, at least for LBP individuals. In other words, patients with acute LBP move the cervical spine more, probably due to their lumbar mobility restrictions and pain, while patients with acute NP move the lumbar spine more, probably due to the mobility restrictions and pain of cervical region.

### 4.2. Capacity of MMPs and ROMs to Discriminate between Spinal Pain and Control Individuals

This is the first study to test the discriminant ability of MMPs to identify patients with acute LBP, acute NP and healthy controls which limits comparisons with previous research. Some specific MMPs and ROM were able to classify subjects between groups according to ROC curves. Interestingly, the only outcome that could discriminate among the three groups was the cervical decrement, and this was also the only variable that achieved acceptable capacity of discrimination (AUC > 0.7). Furthermore, only ROMs in flexion, both in lumbar and cervical regions, could also discriminate among the groups. Again, flexion movement appears to be the most affected in regional spinal pain.

The combination of outcomes to determine their influence on suffering acute NP or LBP, with the control group as reference, determined that two cervical variables, in this case cervical lateral-flexion and cervical decrement, remained in the model when acute LBP and healthy controls were analyzed, and that cervical and lumbar flexions and, again, cervical decrement, remained in the model when acute NP and control groups were analyzed. Therefore, cervical decrement, that is inverse to the elasticity, seems to be the main mechanical property that discriminates groups. Recently, it has been reported that elasticity is lower in elderly patients with chronic LBP [44] and in patients with ankylosing spondylitis [45], although always when assessed in lumbar region, which is consistent with the current results.

Furthermore, the links between cervical and lumbar regions when one of both areas is affected, could be explained by the regional interdependence model, where one region may contribute to, or is associated with, the patient’s primary complaint by different mechanisms, such as biopsychosocial, neurophysiological, or musculoskeletal [75,76]. These patterns increase the relevance for assessing the whole spine as a unique structure.

### 4.3. Associations between MMPs, ROMs with Sociodemographic and Clinical Features

In summary, our results showed a consistent trend, with age being positively correlated with tone, stiffness and relaxation, and negatively correlated with ROM, as occurred with the BMI, in all groups. Furthermore, scant associations were found between MMPs and ROMs with pain, fear, disability and quality of life, in both spinal pain groups.

The association of age with the reduction of ROM has been described in the literature [39], based on structural changes, such as degenerative alterations and soft tissue adaptative shortenings [77]. With respect to MMPs, this relationship has been described in healthy subjects [78] and could be explained by the loss of skeletal muscle mass and strength that occurs with advancing age [79], which increase the interest of these assessments as part of the clinical evaluation of spinal pain individuals.

Controversial results have been reported for the association between MMPs and ROM, and pain in chronic LBP patients, with some data supporting positive correlations with tone, stiffness and decrement [44], whereas others do not [71]. As occurred with the differences among the groups, it is possible that the association between pain and MMPs only appears in the chronic state, as consequence of an interaction or confluence of various predictors such as emotional, cognitive, social and physical factors [39], as described for other musculoskeletal complaints [58], but not in the acute state.

### 4.4. Strengths and Limitations

One of the strengths of the study is its clinical applicability. In fact, the quantification of muscle stiffness of lumbar tissues is of high value for LBP management [45,80], and can assist diagnosis and treatments [80,81] in clinical setting. Thus, the current research has extended the study of MMPs to the acute stages of LBP and NP. Finally, a multivariate analysis was performed to identify variables able to discriminate individuals according to their clinical state.

Nevertheless, a set of limitations should be recognized. First, there was no follow-up period, which prevents any cause–effect relationship between outcomes and clinical state. Second, the applicability of these results is limited to similar population characteristics, in terms of acute and moderate pain, age or BMI. Third, for a better approach to these common conditions in clinical setting, no evaluation of the contraction state at rest of the spinal musculature, such as electromyography (EMG), was performed to confirm the absence of contractions along the MMPs assessment [43]. Furthermore, it has been stated that myotonometry cannot determine the MMPs at more than 2 cm deep [29]. This can be the cause of the lack of lumbar MMPs as discriminators between groups, since myotonometry probably could not obtain data from deep lumbar muscles in many individuals. Fourth, the assessors were not blinded to the individual condition, but the procedures have shown low dependence of the assessor, which reduces their influence in the results. Finally, other techniques for analyzing muscle features, such as surface electromyography (sEMG) [82] and high density EMG [83], could add different information to the data obtained in the current study. There are also other systems for measuring lumbar and cervical mobility, especially motion capture systems [48] and some dedicated mobility machines such as MedX (Ocala, FL, USA) [84], although the feasibility of these systems is low due to the need for expensive dedicated laboratories. Further studies with prospective designs, chronic syndromes, and different assessment protocols should be conducted to improve knowledge in this area.

## 5. Conclusions

The presence of acute LBP and acute NP can increase tone and decrease elasticity of posterior cervical muscles, and modify ROMs in flexion and rotation, which increases the relevance of assessing these features for spinal pain syndromes in clinical settings.

The tissue elasticity discriminates spinal pain individuals from controls. The ROMs in flexion can also help in discriminating between acute pain and pain-free subjects.

Finally, the MMPs and ROMs show a pattern of association with age and BMI in acute spinal pain, but not with intensity and quality of pain, or disability, probably due to the short period of time (acute state) during which pain is suffered.

## Figures and Tables

**Table 1:** Sociodemographic and clinical characteristics of patients with acute low back pain, acute neck pain and healthy controls.

Variables	Low Back Pain (*n* = 33)	Neck Pain (*n* = 33)	Healthy (*n* = 33)	*p*-Value
Age (years)	41.9 ± 14.8	38.8 ± 11.1	37.0 ± 10.9	0.373
Sex (female/male)	11/22	14/19	13/20	0.742
BMI (Kg/m^2^)	25.9 ± 3.9	25.2 ± 4.7	23.8 ± 3.5	0.160
PCS-12	41.6 ± 8.6	42.5 ± 9.9	54.1 ± 3.7	<0.001 ‡
MCS-12	50.9 ± 9.5	50.8 ± 10.4	53.1 ± 6.4	0.484
NPRS	4.7 ± 1.6	5.4 ± 1.9	-	0.100
NWC	9.3 ± 4.9	9.1 ± 4.5	-	0.855
PRI-total	17.1 ± 9.5	18.4 ± 9.0	-	0.561
NDI	-	11.0 ± 5.2	-	
ODI	21.1 ± 12.8	-	-	
FABQ	31.4 ± 13.6	36.2 ± 20.7	-	0.283
FABQ-PA	12.3 ± 6.0	12.6 ± 6.7	-	0.815
FABQ-W	11.8 ± 7.4	16.1 ± 10.7	-	0.072
TSK-11SV	22.9 ± 6.5	22.7 ± 5.9	-	0.891
**Muscle Mechanical Properties (MMPs)**				
Lumbar tone (Hz)	14.94 ± 2.54	14.70 ± 1.63	15.16 ± 2.22	0.697
Lumbar stiffness (N/m)	289.89 ± 76.23	279.95 ± 69.16	283.72 ± 75.37	0.847
Lumbar decrement	1.41 ± 0.37	1.28 ± 0.35	1.26 ± 0.32	0.169
Lumbar relaxation (ms)	19.45 ± 4.59	19.53 ± 4.24	19.48 ± 4.62	0.998
Lumbar creep (Deborah number)	1.21 ± 0.27	1.15 ± 0.23	1.21 ± 0.24	0.519
Cervical tone (Hz)	15.86 ± 2.09	16.52 ± 1.78	15.42 ± 2.24	0.048 §
Cervical stiffness (N/m)	275.92 ± 57.19	290.43 ± 53.97	265.14 ± 72.17	0.258
Cervical decrement	1.43 ± 0.22	1.45 ± 0.18	1.27 ± 0.23	0.001 ‡
Cervical relaxation (ms)	19.62 ± 3.87	18.10 ± 2.48	19.17 ± 4.08	0.214
Cervical creep (Deborah number)	1.17 ± 0.20	1.11 ± 0.13	1.15 ± 0.20	0.312
**Spinal Mobility (Range of Motion, ROM)**				
Lumbar flexion (°)	49.0 ± 14.3	57.1 ± 12.6	53.8 ± 9.4	0.031 *
Lumbar extension (°)	18.6 ± 18.2	17.2 ± 10.5	16.9 ± 11.9	0.871
Lumbar rotation (°)	27.2 ± 11.8	31.0 ± 10.0	27.9 ± 8.5	0.279
Lumbar lateral-flexion (°)	54.7 ± 13.5	55.5 ± 8.8	56.5 ± 9.8	0.807
Cervical flexion (°)	51.8 ± 8.8	46.0 ± 9.9	51.8 ± 7.4	0.010 †
Cervical extension (°)	45.4 ± 11.7	45.2 ± 16.1	50.5 ± 13.0	0.209
Cervical rotation (°)	135.3 ± 19.5	127.8 ± 25.2	139.9 ± 15.0	0.047 §
Cervical lateral-flexion (°)	73.9 ± 19.6	68.6 ± 17.2	69.6 ± 11.4	0.383

§: Statistical differences between acute NP and control groups. ‡: Statistical differences between both acute LBP and acute NP groups against the control group. *: Statistical differences between acute LBP and acute NP groups. †: Statistical differences between acute NP and both LBP and control groups. Abbreviations: BMI: body mass index; NWC: Number of words chosen; NPRS: Numerical pain rating scale; PCS-12: Physical Component Summary of 12-item Short-Form Health Survey; MCS-12: Mental Component Summary of 12-item Short-Form Health Survey; ODI: Oswestry Disability Index; FABQ: Fear-Avoidance Beliefs Questionnaire; FABQ-PA: Physical Activity Subscale of Fear-Avoidance Questionnaire; FABQ-W: Work Subscale of Fear-Avoidance Questionnaire; TSK-11SV: Short Version of Tampa Scale for Kinesiophobia; PRI: Pain Rating Index.

**Table 2:** Identification of clinical state according to multinomial regression model.

State	Group	Estimated State			
		Low Back Pain	Neck Pain	Control	Percentage of Correct Estimation
Real state	Low Back Pain	19	6	8	57.6%
	Neck Pain	7	23	2	71.9%
	Control	5	4	24	72.7%
	Global percentage	31.6%	33.7%	34.7%	67.3%

**Table 3:** Correlations between sociodemographic and clinical characteristics within the acute low back pain group.

Variables	Lumbar Tone	Lumbar Stiffness	Lumbar Decrement	Lumbar Relaxation	Lumbar Creep	Cervical Tone	Cervical Stiffness	Cervical Decrement	Cervical Relaxation	Cervical Creep	Lumbar Flexion	Lumbar Extension	Lumbar Rotation	Lumbar Lateral-Flexion	Cervical Flexion	Cervical Extension	Cervical Rotation	Cervical Lateral-Flexion
Age	0.554	0.555	0.565	−0.455	NS	0.342	NS	0.687	NS	NS	−0.547	NS	NS	−0.397	−0.545	−0.460	−0.352	−0.533
BMI	NS	NS	NS	NS	NS	−0.416	−0.436	NS	0.468	0.455	NS	NS	NS	NS	NS	−0.421	NS	−0.400
Lumbar flexion	−0.372	−0.359	NS	NS	NS	NS	NS	−0.399	NS	NS								
Lumbar extension	NS	NS	NS	NS	NS	NS	NS	NS	NS	NS								
Lumbar rotation	NS	NS	NS	NS	NS	NS	NS	NS	NS	NS								
Lumbar lateral-flexion	NS	NS	NS	NS	NS	NS	NS	NS	NS	NS								
Cervical flexion	NS	NS	NS	NS	NS	NS	NS	NS	NS	NS								
Cervical extension	NS	NS	NS	NS	NS	NS	NS	NS	NS	NS								
Cervical rotation	−0.529	−0.534	−0.386	0.405	0.352	NS	NS	NS	NS	NS								
Cervical lateral-flexion	NS	NS	NS	NS	NS	NS	NS	NS	NS	NS								

Abbreviations: BMI: body mass index; NS: Not significant (*p*-value > 0.05).

**Table 4:** Correlations between sociodemographic and clinical characteristics within the acute neck pain group.

Variables	Lumbar Tone	Lumbar Stiffness	Lumbar Decrement	Lumbar Relaxation	Lumbar Creep	Cervical Tone	Cervical Stiffness	Cervical Decrement	Cervical Relaxation	Cervical Creep	Lumbar Flexion	Lumbar Extension	Lumbar Rotation	Lumbar Lateral-Flexion	Cervical Flexion	Cervical Extension	Cervical Rotation	Cervical Lateral-Flexion
Age	NS	NS	0.458	NS	NS	NS	NS	0.541	NS	NS	−0.459	NS	NS	−0.401	NS	−0.512	−0.483	−0.674
BMI	NS	NS	NS	0.351	0.379	NS	NS	NS	NS	0.341	−0.388	NS	NS	−0.423	−0.403	−0.513	NS	NS
Lumbar flexion	NS	NS	NS	NS	NS	NS	NS	NS	NS	NS								
Lumbar extension	NS	NS	NS	NS	NS	NS	NS	NS	NS	NS								
Lumbar rotation	NS	NS	NS	NS	NS	NS	NS	NS	NS	NS								
Lumbar lateral-flexion	NS	NS	−0.545	NS	−0.348	NS	NS	NS	NS	NS								
Cervical flexion	NS	NS	NS	NS	−0.409	NS	NS	NS	NS	NS								
Cervical extension	NS	NS	NS	NS	NS	NS	NS	NS	NS	NS								
Cervical rotation	NS	NS	NS	NS	NS	NS	NS	NS	NS	NS								
Cervical lateral-flexion	NS	NS	−0.522	NS	NS	NS	NS	−0.364	NS	NS								

Abbreviations: BMI: body mass index; NS: Not significant (*p*-value > 0.05).

**Table 5:** Correlations between sociodemographic and clinical characteristics within the healthy control group.

Variables	Lumbar Tone	Lumbar Stiffness	Lumbar Decrement	Lumbar Relaxation	Lumbar Creep	Cervical Tone	Cervical Stiffness	Cervical Decrement	Cervical Relaxation	Cervical Creep	Lumbar Flexion	Lumbar Extension	Lumbar Rotation	Lumbar Lateral-Flexion	Cervical Flexion	Cervical Extension	Cervical Rotation	Cervical Lateral-Flexion
Age	0.644	0.598	0.653	−0.629	−0.410	NS	NS	0.529	NS	NS	−0.376	−0.650	NS	NS	NS	−0.354	NS	NS
BMI	NS	NS	NS	NS	NS	NS	NS	NS	0.494	0.510	−0.367	NS	NS	−0.459	NS	−0.502	NS	NS
Lumbar flexion	−0.411	−0.341	−0.384	0.413	0.341	NS	NS	NS	NS	NS								
Lumbar extension	−0.354	−0.381	−0.490	0.629	0.570	NS	NS	−0.401	NS	NS								
Lumbar rotation	NS	NS	NS	NS	NS	NS	NS	NS	NS	NS								
Lumbar lateral-flexion	NS	NS	NS	NS	NS	NS	NS	NS	NS	NS								
Cervical flexion	NS	NS	NS	NS	NS	NS	NS	NS	NS	NS								
Cervical extension	NS	NS	NS	NS	NS	NS	NS	NS	NS	−0.352								
Cervical rotation	NS	NS	NS	NS	NS	NS	NS	NS	NS	NS								
Cervical lateral-flexion	NS	NS	−0.464	NS	NS	NS	NS	NS	NS	NS								

Abbreviations: BMI: body mass index; NS: Not significant (*p*-value > 0.05).

## Abbreviations

AUC	Area Under the Curve
BMI	Body Mass Index
CV	Coefficient of Variation
EMG	Electromyography
sEMG	surface Electromyography
FABQ	Fear-Avoidance Beliefs Questionnaire
FABQ-PA	Physical Activity Subscale of Fear-Avoidance Beliefs Questionnaire
FABQ-W	Work Subscale of Fear-Avoidance Beliefs Questionnaire
ICC	Intraclass Correlation Coefficient
IMUs	Inertial Motion Units
LBP	Low Back Pain
MMP	Muscle Mechanical Property
NDI	Neck Disability Index
NP	Neck Pain
NPRS	Numerical Pain Rating Scale
NS	Not Significant
NWC	Number of Words Chosen
ODI	Oswestry Disability Index
OR	Odds Ratio
PRI	Pain Rating Index
ROC	Receiver Operating Characteristic
ROM	Range of Motion
SF-12	12-item Short-Form Health Survey
MCS-12	Mental Component Summary of 12-item Short-Form Health Survey
PCS-12	Physical Component Summary of 12-item Short-Form Health Survey
TSK-11SV	Short Version of Tampa Scale for Kinesiophobia
95%IC	95% confidence interval
